# *Bombyx mori* Dihydroorotate Dehydrogenase: Knockdown Inhibits Cell Growth and Proliferation via Inducing Cell Cycle Arrest

**DOI:** 10.3390/ijms19092581

**Published:** 2018-08-30

**Authors:** Erhu Zhao, Xiaolan Jiang, Hongjuan Cui

**Affiliations:** 1 State Key Laboratory of Silkworm Genome Biology, College of Biotechnology, Southwest University, Chongqing 400716, China; erhuzhao@126.com (E.Z.); xiaolan.j@hotmail.com (X.J.); 2 Chongqing Engineering and Technology Research Center for Silk Biomaterials and Regenerative Medicine, Chongqing 400716, China; 3 Southwest University Engineering Research Center for Cancer Biomedical and Translational Medicine, Southwest University, Chongqing 400715, China

**Keywords:** BmDHODH, leflunomide, cell proliferation, cell cycle arrest, DNA replication

## Abstract

Dihydroorotate dehydrogenase (DHODH), in the de novo pyrimidine biosynthetic pathway, is the fourth enzyme of pyrimidine synthesis and is used to oxidize dihydroorotate and hence to orotat. We cloned and characterized here the *dhod* of silkworms, *Bombyx mori*. The full-length cDNA sequence of *dhod* is 1339 bp, including an open reading frame (ORF) of 1173 bp that encoded a 390 amino acid protein. And two domains were involved in the Dihydroorotate dehydrogenase amino acid sequence of silkworms, *Bombyx mori* (BmDHODH), namely a DHO_dh domain and a transmembrane domain in N-termina. The silkworm *dhod* is expressed throughout development and in nine tissues. Moreover, knockdown of the silkworm *dhod* gene reduced cell growth and proliferation through G2/M phase cell cycle arrest. Similarly, DHODH inhibitor (leflunomide) also reduced cell growth and proliferation, with a significant decrease of *cyclin B* and *cdk2*. DHODH is the fourth enzyme of pyrimidine synthesis, so we also found that leflunomide can inhibit, at least in part, the endomitotic DNA replication in silk glands cells. These findings demonstrate that downregulation of BmDHODH inhibits cell growth and proliferation in silkworm cells, and the endomitotic DNA replication in silk gland cells.

## 1. Introduction

Pyrimidine nucleotide synthesis is an essential biological process, which play a pivotal role in cellular metabolism serving as activated precursors of RNA and DNA [1]. This process is conducted by both de novo and salvage pathways. Dihydroorotate dehydrogenase (DHODH) is the fourth enzyme in the de novo pyrimidine biosynthesis pathway, serving as the catalyst to oxidize the dihydroorotate to orotic acid in the biosynthesis of uridine monophosphate (UMP) [2,3]. This acid is the essential precursor of all other pyrimidine nucleotides. The impairment of DHODH activity causes cell growth retardation and cell cycle arrest. This is considered to result from a block in progression from G2/M phase, but not from impaired DNA synthesis induced by a pyrimidine deficiency [4,5,6]. In human, DHODH has been validated as a potential target for treating a variety of autoimmune disease as well as cancer [7]. In malaria parasite, *Plasmodium falciparum*, DHODH has been recognized as a latent drug target to inhibit malarial activity in vivo [8]. The inhibitors on *Pneumocystis jiroveci* (previously called *Pneumocystis carinii*) DHODH are also used to treat *Pneumocystis jiroveci* pneumonia [9,10]. Therefore, DHODH is a high-potential drug target, for example leflunomide, a DHODH specific inhibitor, is used to treat malaria and *Pneumocystis jiroveci* infections as a low molecular weight compound [2,11,12,13]. Leflunomide is also reported to be able to effectively reduce cell growth and proliferation by inhibiting DHODH activity in several types of cancers [14,15,16,17,18]. However, there is little information available on the functions of DHODH in insects. Drosophila melanogaster DHODH has ever been shown with properties common to the other animal DHODHs: mitochondrial localization and electron transport chain coupling via quinones, and its protein sequence strongly resembles the mammalian protein [19,20,21]. Yet further researches are still required. Therefore, the *dhod* gene of silkworm, *Bombyx mori*, was cloned and characterized here.

The silkworm (*Bombyx mori*) is a potential research model for the study of lepidoptera pests biology. Besides, as genetic manipulation technologies have been successfully established, some new tools are provided to identify and characterize more novel genes in silkworm [22,23,24]. In this study, we analyzed the differentiation, description, expression, and functions of the silkworm *dhod* gene. We investigated the role of the *dhod* gene on cell growth and proliferation in the BmE-SWU3 cell line, which was established from silkworm embryos and takes on potent growth vigor and genetic stability [25]. Furthermore, there are multiple endomitotic cell cycles in silk gland cells during larval development [26,27]. In our previous work, we also found that the cell cycles of endomitosis are activated during the intermolt stages and are inhibited during the molt stages in silk gland cells [28]. Given that DHODH is the fourth enzyme of pyrimidine synthesis, we investigated the effects of DHODH inhibitor on endomitotic DNA synthesis in silk glands cells.

## 2. Results

### 2.1. Cloning and Characterization of dhod in Silkworm, Bombyx mori

**Figure 1:**
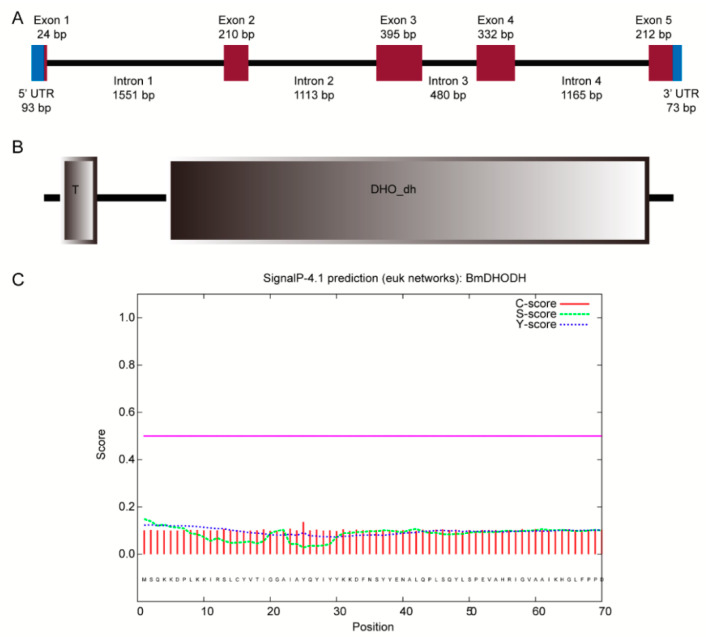
Cloning and characterization of *dhod*, *Bombyx mori*. (**A**) The gene structure of *dhod* in silkworm. Exons and introns are represented by brown box and black solid lines, respectively. The 5’ and 3’ UTRs are represented by blue box. (**B**) The putative structure protein domain of BmDHODH. The domain was predicted by SMART. (**C**) The signal peptide predication of BmDHODH. The result was generated by SignalP 4.1 Server.

The complementary DNA (cDNA) of *dhod* was obtained by amplifying polymerase chain reaction (PCR) and rapid-amplification of cDNA ends (RACE), i.e., the rapid magnification of cDNA ends while the result was verified by amplifying the open reading frames (ORF). The full-length cDNA sequence of *BmDHODH* is 1339 bp. It included a 1173 bp ORF that encoded a 390 aa protein, a 93 bp 5′ UTR, and a 73 bp 3′ UTR (Figure 1A), which were entirely clustered on nscaf3032 situated on chromosome 26 in silkworm genome. Two domains were involved in the *BmDHODH* aa sequence, namely a DHO_dh domain and a transmembrane domain in N-terminal (Figure 1B and Figure S1). Besides, SignalP 4.1 was employed to get the advance information of the position and orientation of the signal peptide cleavage sites in *dhod* sequence for assuming about potential signal peptides of BmDHODH proteins. The Y-score from the SignalP output was adopted to discriminate the signal and nonsignal peptide. As shown in Figure 1C, the BmDHODH sequence contains no cleavage site, which means it belongs to a nonsecretory protein.

### 2.2. Phylogenetic Analysis of DHODH Homologues

**Figure 2:**
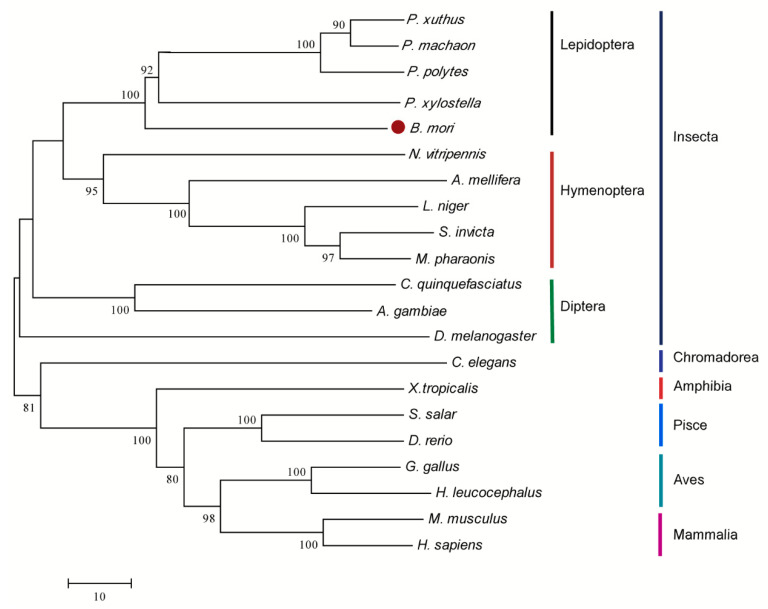
Phylogenetic analysis of *DHODH* homologues. The phylogenetic tree of *DHODHs* were established by neighbor-joining method. The number closed to individual branches represents the percentage of 1000 bootstrap iterations supporting the branch, and values below 60% were omitted.

To explore the evolution of the silkworm and other species, a phylogenetic tree of aligned aa sequences was established from various species using MEGA 6.0. The phylogenetic analysis suggests that *DHODH* was conserved from invertebrates and vertebrates. Yet the members can still be classified into two types: vertebrates (including Mammalia, Aves, Pisce, and Amhibia) and invertebrates (Insecta). Insecta can also fall into three subgroups: Lepidoptera, Hymenoptera, and Diptera (Figure 2). Expectedly, silkworm *dhod* is clustered into Lepidoptera subgroups; it is the most closely associated with ones of *Plutella xylostella*, *Papilio polytes*, *Papilio machaon*, and *Papilio xuthus*, which form a clade (Figure 2).

### 2.3. Amino Acid Sequence Alignment of BmDHODH Homologues 

**Figure 3:**
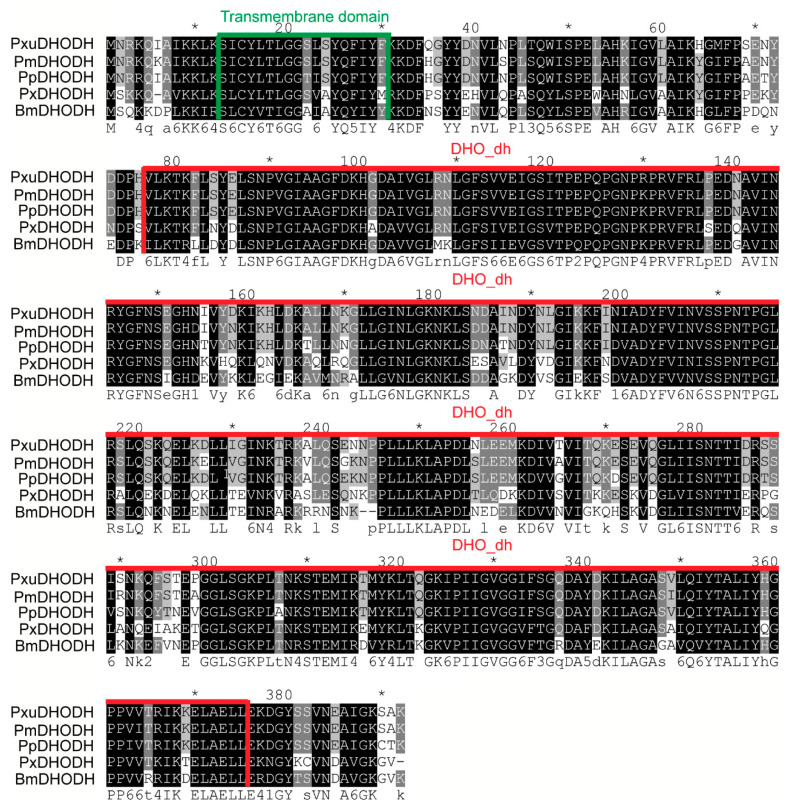
Phylogenetic analysis of DHODH homologues. Multiple alignment of DHODH aa sequences from *Plutella xylostella* (PxDHODH, XP_011556582.1), *Papilio polytes* (PpDHODH, XP_013138102.1), *Papilio machaon* (PmDHODH, XP_014361985.1), and *Papilio xuthus* (PxuDHODH, KPJ04273.1). Identical amino acids and those shared in more than four sequences are highlighted by black and gray, respectively. The DHO_dh motif is underlined in red. The transmembrane domain is underlined in green, which was predicted by SMART (http://smart.embl-heidelberg.de/).

The homology of BmDHODH and other species DHODH sequences were explored through multiple sequence alignment using ClustalX. As suggested in the results, BmDHODH is shared with PxDHODH, PpDHODH, PmDHODH, and PxuDHODH in 70%, 67%, 67%, and 67%, respectively. These sequences shared much similarity to BmDHODH sequence and contained a highly conserved motif, the DHO_dh motif (Figure 3). The secondary structural analysis of the homologues suggests that all homologues contained a conceivable N-terminal transmembrane domain and their aa locations were 12–29 (PxDHODH) and 13–30 (BmDHODH, PpDHODH, PxuDHODH, PmDHODH) (Figure 3).

### 2.4. The Expression Profile of dhod in Silkworm, Bombyx mori

**Figure 4:**
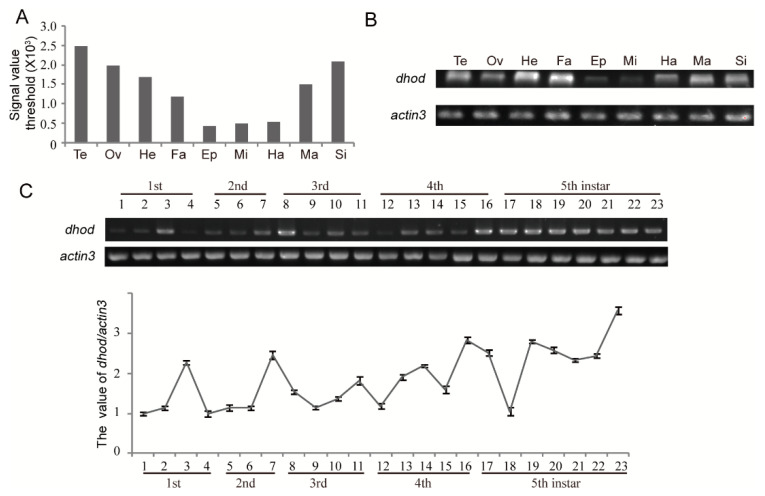
The expression profile of *dhod*, *Bombyx mori*. (**A**) The expression levels of *dhod* were generated from 3-day-old 5th instar larval tissues based on EST database of silkworm. (**B**) The expression levels of *dhod* were achieved from 3-day-old 5th instar larval tissues by RT-PCR, and *actin3* was used as a control. A and B: Te: Testis; Ov: Ovary; He: Head; Fa: Fat body; Ep: Epidermis; Mi: Midgut; Ha: Hemolymph; Ma: Malpighian tubles; Si: Silk gland. (**C**) The expression profile of *dhod* was performed by RT-PCR during whole larvae development stages in silkworm, and *actin3* was used as a control. 1–3: 1st instar day 1 to day 3; 4: 1st instar premolting; 5–6: 2nd instar day 1 to day 2; 7: 2nd instar premolting; 8–10: 3rd instar day 1 to day 3; 11: 3rd instar premolting; 12–15: 4th instar day 1 to day 4; 16: 4th instar premolting; 17–23: 5th instar day 1 to day 7. Data represent the average ± SD of at least three independent experiments. The value of *dhod* expression was calculated by using Quantity One software.

The expressed-sequence tag (ETS) database from silkworm at stage of 3-day-old 5th instar larvae was analyzed. It was found that *dhod* was highly expressed in several tissues including testis, ovary, head, and silk gland. However, its expression was relatively low in epidermis, mid-gut, and hemolymph while moderate in fat and malpighian tubles (Figure 4A); we confirmed this result by qRT-PCR (Figure 4B). Next, the expression profile of *dhod* was analyzed in different developmental stages. The result illustrated that *dhod* was commonly expressed throughout the entire developmental stages. Generally, the *dhod* expression level in premolting period was greater than that in other corresponding stages (Figure 4C). 

### 2.5. Knockdown of BmDHODH Inhibited Cell Growth and Proliferation through G2/M Cell Cycle Arrest

**Figure 5:**
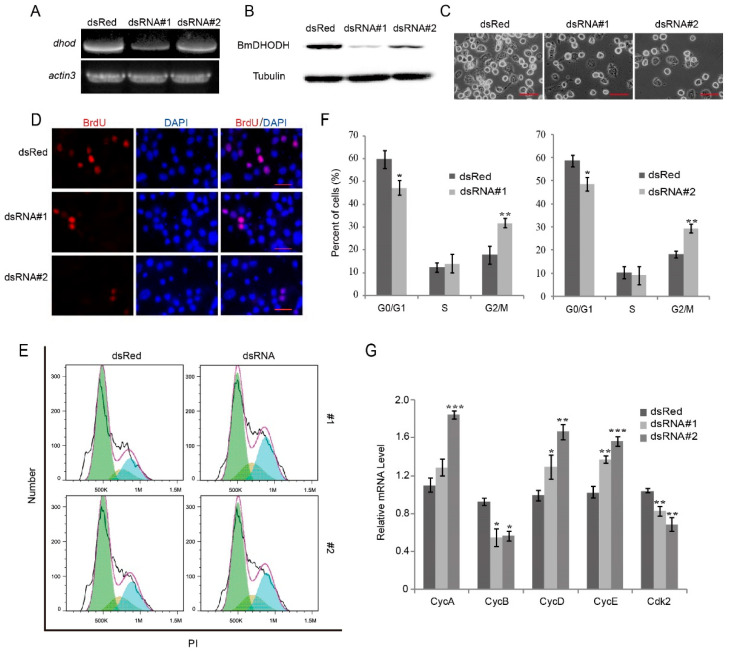
Knockdown of BmDHODH inhibited cell growth and proliferation. (**A**) RT-PCR was performed to detect *dhod* mRNA expression level in BmE-SWU3 cells after knockdown of BmDHODH by dsRNA interference for 48 h; *actin3* was used as a control. (**B**) Western blot assay was performed to detect BmDHODH protein expression level in BmE-SWU3 cells after knockdown of BmDHODH by dsRNA interference for 48 h; tubulin was used as a control. (**C**) Morphologic examination of BmE-SWU3 cells was shown after dsRNA interference for 48 h; dsRed was used as a control. Scale bar, 50 µm. (**D**) Cells were grown on coverslips after dsRNA interference for 48 h, respectively, and dsRed was used as a control. Cells were stained with an antibody against BrdU (red) and counterstained with 4′,6-diamidino-2-phenylindole (DAPI) (blue); scale bar, 100 µm. BrdU-positive cells were calculated randomly in at least 10 fields under microscopy. (**E**,**F**) Cell cycle was analyzed by a FACS assay, after knockdown of BmDHODH by dsRNA interference in BmE-SWU3 cells, and dsRed was used as a control. (**G**) qRT-PCR analysis was performed for BmE-SWU3 cells after dsRNA#1 or dsRNA#2 interference for 48 h, and dsRed was used as a control. In (**F**,**G**), data represent the average ± SD of at least three independent experiments. Statistical analysis was performed using Student’s *t*-test or one-way analysis of variance (ANOVA): * *p* < 0.05, ** *p* < 0.01, and *** *p* < 0.001.

To further explore the function of BmDHODH in the silkworm, DsRNA interference was employed to knock down BmDHODH expression. BmDHODH expressivity was checked by RT-PCR analysis as well as Western blot assay, and dsRed served as a control. As predicted, dsRNA#1 or dsRNA#2 could significantly decrease BmDHODH mRNA and protein level in comparison with dsRed control (Figure 5A,B). Knock down of BmDHODH expression by dsRNA largely inhibited cell proliferation (Figure 5C and Figure S2A). To further investigate whether BmDHODH knockdown change the rate of nuclear DNA synthesis, BrdU staining assay was employed. The result shows that knock down of BmDHODH expression significantly decreased DNA replication (Figure 5D and Figure S2B). Generally, knockdown of BmDHODH expression by dsRNA significantly inhibits cell growth and proliferation in BmE-SWU3 cells. 

To better understand how BmDHODH knockdown inhibits cell growth and proliferation, the cell cycle was analyzed by flow cytometry. As illustrated in Figure 5E,F, knockdown of BmDHODH by dsRNA interference led to the accumulation in G2/M phase in BmE-SWU3 cells. We further examined the molecular mechanism by which BmDHODH knockdown induces G2/M cell cycle arrest. Accordingly, the associated cell cycle regulatory factors were investigated by qRT-PCR, which involved *cyclin A*, *cyclin B*, *cyclin D*, *cyclin E*, and *cdk2.* We found *BmDHODH* knockdown significantly decreased the mRNA level of *cdk2* and *cyclin B* and increased *cyclin A*, *cyclin*, *D* and *cyclin E* (Figure 5G). In conclusion, knockdown of BmDHODH by dsRNA interference led to an accretion of cells at the G2/M stage with the decease of certain cell cycle factors that regulated the proceeding of G2/M phase, and inhibited cell growth and proliferation in BmE-SWU3 cells.

### 2.6. DHODH Inhibitor Both Reduced Cell Growth and Proliferation and DNA Synthesis of Silk Gland Cells 

**Figure 6:**
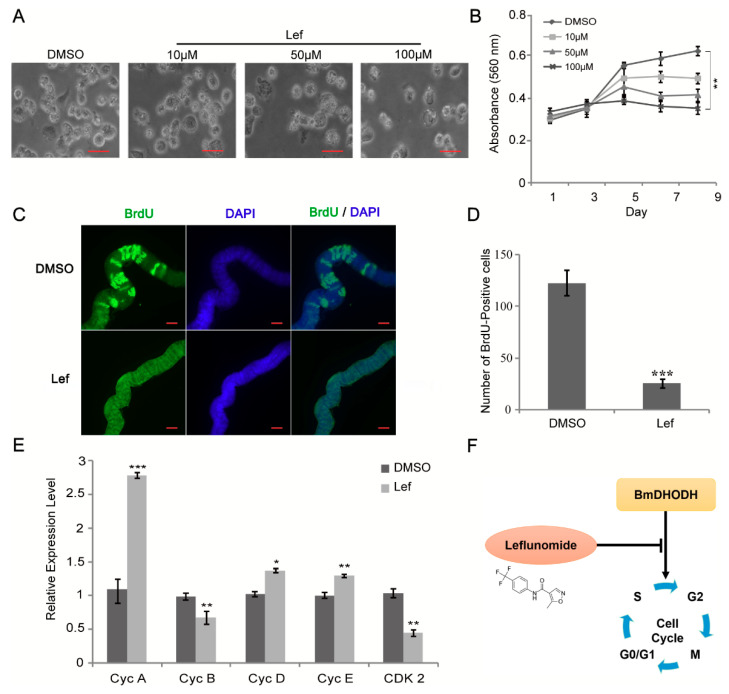
Leflunomide reduced cell growth and proliferation though suppressing the expression of BmDHODH. (**A**) Morphologic examination of BmE-SWU3 cells was showed after treated with 10, 50, and 100 μM leflunomide for 96 h, DMSO was used as a control. Scale bar, 50 μm. (**B**) After treated with 100 μM leflunomide, cell growth was detected every other day by MTT assay, DMSO was used as a control. (**C**,**D**) The analysis of BrdU-labeled cells in each silk gland treated with 100 μM leflunomide for 24 h. DMSO was used as a control. Scale bar, 100 μm. (**E**) The qRT-PCR analysis was performed in BmE-SWU3 cells after treated with 100 μM leflunomide for 96 h. DMSO was used as a control. (**F**) Model for BmDHODH in control of cell cycle progression. The black “T” arrow represents “inhibition”; and the black arrow represents “promotion”. In (**B**,**D**,**E**), each bar represented the average ± SD of three independent experiments. Statistical analysis was performed using the 2-tailed Student’s *t*-test, * *p* < 0.05, ** *p* < 0.01, *** *p* < 0.001.

Leflunomide is capable of inhibiting the enzyme DHODH, thus counting as a DHODH inhibitor. To verify whether leflunomide exerted an impact on cell growth and proliferation, cell morphology was first observed after leflunomide treatment, followed by trypan blue assay. According to the results, it is shown that cell growth was repressed by leflunomide (Figure 6A and Figure S3A). The MTT assay was also performed and presented that cell proliferation was significantly inhibited with leflunomide in comparison with DMSO group (Figure 6B). In addition, BrdU staining assay was employed and BrdU-positive cells in leflunomide treated group were less than DMSO treated group (Figure S3B,C), which suggested that leflunomide significantly suppressed cell growth and proliferation. 

DHODH is the fourth enzyme of pyrimidine synthesis, so we investigate the effects of a DHODH inhibitor on endomitotic DNA synthesis in silk glands cells. The silk glands from 1-day-old 4th instar larvae were dissected and then cultured in Grace medium with or without DHODH inhibitor. As shown in Figure 6C,D, the result showed that DHODH inhibitor decreased significantly the number of BrdU-positive silk gland cells. To further investigate the mechanism of the inhibition by leflunomide, the expression levels of associated cell cycle regulatory factors were checked using qRT-PCR. The results suggest that *cdk2* and *cyclin B* mRNA levels significantly decreased while DMSO-treated cells, and *cyclin A*, *cyclin D*, and *cyclin E* levels increased (Figure 6E). These findings are consistent with the model that knockdown of BmDHODH inhibited cell growth and proliferation through control of cell cycle regulatory factors. Together, these results demonstrate that BmDHODH regulates cell cycle progression in BmE-SWU3 cells (Figure 6F).

## 3. Discussion

DHODH is a critical biological enzyme closely correlated with cell growth and proliferation. Previous studies suggested that the decrease of DHODH activity is associated with mitochondrial dysfunction, and causes cell growth retardation and cell cycle arrest in mammal models [6,29,30]. Loffler et al. first studied and characterized a purified insect dihydroorotate dehydrogenase [19]. They found the Drosophila DHODH is very similar to its mammalian forms in its catalytic kinetic properties, but significantly different in its sensitivity to diverse inhibitors previously evaluated for inhibition of mammalian DHODHs. Furthermore, they confirmed that the prodrug leflunomide with its active metabolite was the most effective agents from the study on the insect DHODH. But besides that, relatively little is known about the insect DHODH.

In this study, we employed a bioinformatic approach to identify BmDHODH based on the completion of silkworm genome sequencing [31,32,33]. The *dhod* gene of the silkworm was further cloned and characterized using PCR amplification and the RACE method. The BmDHODH amino acid sequence contained a DHO_dh domain and a transmembrane domain in N-terminal, which was conserved from invertebrates and vertebrates. And the *dhod* was commonly expressed in all silkworm larval stages of silkworm and peaked in premolting period, which indicated that *dhod* may correlate with higher metabolism rate in the molting period. Furthermore, dsRNA interference was found able to inhibit cell growth and proliferation. It made the cell cycle arrested at the G2/M stage, with a significant decrease of *cyclin B* and *cdk2*, which are the determinants of G2/M transition in the cell cycle [34,35]. These results are consistent with previous reports that DHODH knockdown induces cell growth retardation and G2/M cell cycle arrest in mammalian models [6]. Generally, these findings also indicate that BmDHODH controls the expression of several genes such as *cyclin B* and *cdk2*, which are involved in silkworm cell proliferation. 

There are two domains in human *DHODH* gene, including an alpha-helical domain, which forms the opening of the tunnel guiding to the active site, and an alpha/beta-barrel domain in which the active site is located [30,36]. It is proven that leflunomide can bind in this tunnel, which serves as a specific inhibitor of DHODH [30]. Similarly, leflunomide also reduced cell growth and proliferation, with a significant decrease of *cyclin B* and *cdk2*. Furthermore, DHODH is the fourth enzyme of pyrimidine synthesis, so we found that leflunomide can inhibit, at least in part, the endomitotic DNA replication in silk glands cells. These findings, together with those presented above, demonstrate that downregulation of BmDHODH inhibits cell growth and proliferation, and the endomitotic DNA replication in silk glands cells. However, more researches with silkworms are still needed, which could uncover very interesting BmDHODH properties. Our work might be a promising tool to help researchers understand the characteristics of BmDHODH. Furthermore, pyrimidine biosynthesis is an essential process for DNA and RNA synthesis in silkworm. A critical step in the de novo pyrimidine biosynthesis pathway is catalyzed by BmDHODH. Therefore, BmDHODH is a potential drug target. The silkworm is a promising lepidoptera pest model, so the search for inhibitors of DHODH may provide new insights into ecofriendly pest control.

## 4. Experimental Section

### 4.1. Biological Materials 

This study adopted the Chinese silkworm strain Dazao (P50) and kept in the State Key Laboratory of Silkworm Genome Biology. The food of the grub were artificial diet or fresh mulberry leaves at 25 ± 2 °C, following a cycle of 12 h light/12 h dark. In such development period, the specimens would be isolated from the larval and stored in liquid nitrogen before being adopted. The BmE-SWU3 cell line [25] was cultured in Grace medium (GIBCO BRL, Gaithersburg, MD, USA) at 27 °C supplemented with 10% fetal bovine serum (FBS; Invitrogen, Carlsbad, CA, USA) and 1% penicillin-streptomycin (P/S) in 25 cm^2^ T-flasks. The medium was changed every three days.

### 4.2. Prediction of BmDHODH and Full-Length cDNA Cloning

The bioinformatics method following CDS database, EST database and silkworm genome was employed to identify silkworm *dhod* and predicted the protein database of *Bombyx mori* (http://www.silkdb.org/silkdb/). The other amino acid (aa) sequences were acquired from the NCBI GenBank (http://www.ncbi.nlm.nih.gov/). Additionally, *DHODH* sequences of some other species were utilized to investigate BLAST against the silkDB via an E-value initiation point 10^−6^ [37,38]. As a result, the domain predication validated every putative protein via SMART (http://smart.embl-heidelberg.de/). 

PCR works in acquiring the segments of silkworm *dhod*, and the forecasted CDS and EST sequences within the SilkDB could help the design of the primers. As a result, the 3′ and 5′ RNA ligase-mediated speedy expansions were carried out in the cDNA ends to acquire the entire length of the cDNA using a GeneRacer^TM^ kit (Invitrogen, Carlsbad, CA, USA) with the gene-specific primer. Eventually, every ORF (open reading frame) was confirmed by PCR. In the next stage, the entire PCR products were cloned into the PMD19-T Simple vector in the Japanese TaKaRa company as well as the sequence of the PCR products in the Invitrogen Corporation in Shanghai, China.

### 4.3. Bioinformatic Analysis

The ORF of *dhod* in silkworm was ascertained by the ORF Finder software (http://www.ncbi.nlm.nih.gov/gorf.html). Besides, the signal peptide was predicted using the SignalP 4.1 (http://www.cbs.dtu.dk/services/SignalP). SMART was employed to predict the domain (http://smart.embl-heidelberg.de/). Moreover, the ClustalX program worked in aligning the aa sequences of BmDHODH, and the neighbor-joining method was employed to establish the BmDHODH with 1000 bootstrap replicates using the MEGA 6.0 program [39]. 

### 4.4. The dsRNA Production

Three DNA fragments with the length of approximately 473, 460, and 394 bp were amplified by PCR. Primers were listed in Table 1. MEGAScript^TM^ RNAi Kit (Ambion, Austin, TX, USA) was employed to synthesize dsRNAs; the PCR products served as templates. Three groups of dsRNA were generated and analyzed by 1% agarose gel electrophoresis to ensure their purity, dsRed served as control, and spectrophotometry was used to check the concentration of dsRNAs.

### 4.5. Drug Treatment and Transfection

Leflunomide (Sigma, St. Louis, MO, USA) was dissolved in dimethyl sulfoxide (DMSO) as 200 mM stock solutions. BmE-SWU3 cells were treated with leflunomide (10, 50, and 100 μM) for 96 h. DMSO served as a control in this process. Cell survival was analyzed by trypan blue exclusion assay. Two sets of dsRNA were employed to knock down BmDHODH, and dsRed served as a control. Cells were diluted with fresh medium and seeded 2 × 10^5^ cells into 24-well culture plates the day before transfection. Serum-free transfection with dsRNA was performed using the TransMessenger^TM^ transfection Reagent (QIAGEN, Hilden, Germany) following the description of the manufacturer. 

### 4.6. Cell Proliferation Assay

Cell proliferation was measured using the (3-(4,5-dimethylthiazol-2-yl)-2,5-diphenyl) tetrazolium bromide (MTT) assay (Sigma, USA). Cell loading was realized when cells were planted into 96-well plates with density of 8 × 10^3^ cells per well. A microplate reader was employed to measure the absorbance values at at 560 nm. Notably, each experiment was performed respectively at least three occasions.

### 4.7. BrdU Staining

A primary rat antibody against BrdU (Abcam, Cambridge, MA, USA) was employed to incubate the cells for 1 h, which were preconditioned with 10 µM thymidine analog 5-bromo-2-deoxyuridine (BrdU) (Sigma, USA) for 2 h. It is an appropriate secondary antibody for 1 h. The 300 nM 4′,6-diamidino-2-phenylindole (DAPI) (Beyotime, Shanghai, China) was added for counterstaining. In the florescence mounting medium (Beyotime), the cells were first mounted and then examined using an Image-Pro Plus software of a Nikon microscope for the analysis of the images. 

### 4.8. Cell Cycle Assay

The targeted cells were fixed in 70% ethanol after 96 h treatment, and then stained with propidium iodide (PI) (Beyotime, China). The FACScan instrument (BD BioSciences, Ann Arbor, MI, USA) was adopted to pass through the cells, and the CellQuest analysis software was applied for data analysis. 

### 4.9. Western Blot Assay

SDS-PAGE with corresponding gel concentration was adopted to fractionate the proteins isolated with RIPA lysis buffer. Antibody against tubulin and BmDHODH (prepared from Zoonbio Biotechnology, Nanjing, China) were purchased from Beyotime. The BmDHODH expression value was measured using the Quantity One Software (Version 4.6.8).

### 4.10. Effects of Leflunomide on DNA Synthesis in Silk Gland Cells

Culture of silk glands was performed in vitro as described previously [26,28,40]. Silk glands from 1-day-old 4th instar larvae were incubated with or without 100 μM leflunomide for 24 h. Subsequently, the silk glands incubated in Grace’s insect cell culture medium containing 15 μM BrdU for 1 h. The number of BrdU-positive cells was determined.

### 4.11. RNA Extraction and RT-PCR and qRT-PCR Analysis

The TRIzol Reagent (Invitrogen Life Technologies, Carlsbad, CA, USA) was employed to extract the RNA following the manufacturer’s protocol. Each sample was then transcribed reversely. Consequently, the cDNA was amplified. The semiquantitative RT-PCR amplification (RT-PCR) was performed under the following conditions. First and foremost, it should be exposed at 94 °C for a period of 5 min. Subsequently, 30 cycles were performed at 94 °C for 30 s. Later, it was posed at 55.5 °C for 30 s while 72 °C for 1 min, and extended at 72 °C for 10 min. The expression of housekeeping gene *actin3* was analyzed using Quantity One software. And the corresponding expressivity was measured after the standardization following the quantification of *actin3* mRNA expression. Table 2 below lists the primers used in the research. 

SYBR^®^ Premix Ex Taq^TM^ II (TaKaRa, Shiga, Japan) as well as a StepOnePlus^TM^ Real-Time PCR system (Applied Biosystems, Foster City, CA, USA) worked in the process of Quantitative real-time PCR (qRT-PCR). The conditions for the PCR were being exposed at 95 °C for 30 s, followed by 40 cycles at 95 °C for 5 s and 60 °C for 30 s. Table 2 below lists all the primers adopted by each gene. *Actin3* served as a control. The corresponding gene expressivity was computed following the 2^−ΔΔCt^ method [41].

### 4.12. Statistical Analysis

Assays were performed in triplicates. The analysis results were presented as mean ± S.D. The two-tailed *t*-tests played a role in verifying the difference between distinct treatment groups. According to the result, *p* is less than 0.05, which was of statistical significance.

## Figures and Tables

**Table 1:** The Primers of dsRNA.

Name	Primer	Sequence
dsRNA#1	dsRNA#1-F	GTAATACGACTCACTATAGGGAGAACAACTACAATGTCGCAGAA
	dsRNA#1-R	GTAATACGACTCACTATAGGGAGATTCGTCGTGCCCTATGCT
dsRNA#2	dsRNA#2-F	GTAATACGACTCACTATAGGGAGATCGACAAGCACGGAGAC
	dsRNA#2-R	GTAATACGACTCACTATAGGGAGAAGCCAGTTTGAGGAGCAG
dsRed	dsRed-F	GTAATACGACTCACTATAGGGAGAATGGTGAGCAAGGGCGA
	dsRed-R	GTAATACGACTCACTATAGGGAGATTACTTGTACAGCTCGTCCATG

**Table 2:** The primers of Related Genes in Silkworm.

Gene Name	Primer Name	Sequence
*dhod*	dhod-F	GGCTTCAACAGCATAGGGC
	dhod-R	CAGCCACATCCGAGAACTTTT
*cyclin A*	CycA-F	CTCTCAACACCCACCTCAC
	CycA-R	CGCTGCTATTACTGAGGGT
*cyclin B*	CycB-F	TTGCGAGACCGATACCTTTG
	CycB-R	AGATTGCTGCCGCTGCTA
*cyclin D*	CycD-F	CCTCAAAGTTTCGTCAGTGTCATC
	CycD-R	GCATAATCTCCCATTGCCTCA
*cyclin E*	CycE-F	CCCAAGACAATCCAGGCAA
	CycE-R	AGAGGCGAGTCCACCCCA
*cdk 2*	Cdk2-F	GGTACACCAGGCGAGGCACTATG
	Cdk2-R	CACCAGAGTCGCATCAGCCAAG
*actin3*	actin3-F	CGGCTACTCGTTCACTACC
	actin3-R	CCGTCGGGAAGTTCGTAAG

## Supplementary Materials

Supplementary materials can be found at https://www.mdpi.com/1422-0067/19/9/2581/s1.

## Abbreviations

DHODH	Dihydroorotic dehydrogenase
UMP	Uridine monophosphate
cDNA	The complementary DNA
ORF	The open reading frames
RACE	Rapid-amplification of cDNA ends
PCR	Polymerase chain reaction
RT-PCR	Real time-Polymerase chain reaction
qRT-PCR	Quantitative real time-Polymerase chain reaction
Lef	Leflunomide
BrdU	5-bromo-2-deoxyuridine
DAPI	4′,6-diamidino-2-phenylindole
FBS	Fetal bovine serum
P/S	Penicillin-streptomycin
CycA	Cyclin A
CycB	Cyclin B
CycD	Cyclin D
CycE	Cyclin E
Cdk2	Cyclin-dependent kinase 2
