# Correction: Zhao et al. *Bombyx mori* Dihydroorotate Dehydrogenase: Knockdown Inhibits Cell Growth and Proliferation via Inducing Cell Cycle Arrest. *Int. J. Mol. Sci.* 2018, *19*, 2581

**DOI:** 10.3390/ijms262411847

**Published:** 2025-12-08

**Authors:** Erhu Zhao, Xiaolan Jiang, Hongjuan Cui

**Affiliations:** 1 State Key Laboratory of Silkworm Genome Biology, College of Biotechnology, Southwest University, Chongqing 400716, China; erhuzhao@126.com (E.Z.); xiaolan.j@hotmail.com (X.J.); 2 Chongqing Engineering and Technology Research Center for Silk Biomaterials and Regenerative Medicine, Chongqing 400716, China; 3 Southwest University Engineering Research Center for Cancer Biomedical and Translational Medicine, Southwest University, Chongqing 400715, China

In the original publication, there were mistakes in Figure 5 as published [1]. In Figure 5A, the gel band of *dhod* was cropped from the wrong gel, and they are now replaced with the correct picture cropped from the original gel band. In Figure 5C, we mixed some images in the knockdown and control groups. The wrong images were corrected and replaced with the original images. Moreover, there was a mistake in the legend for Figure 5. We incorrectly described the statistical method used. The correct Figure 5 appears below.

We confirm that this correction does not change the interpretation of the results or the conclusions of the article. We are deeply sorry for the mistake and sincerely apologize for any inconvenience caused. This correction was approved by the Academic Editor. The original publication has also been updated.

## Figures and Tables

**Figure 5:**
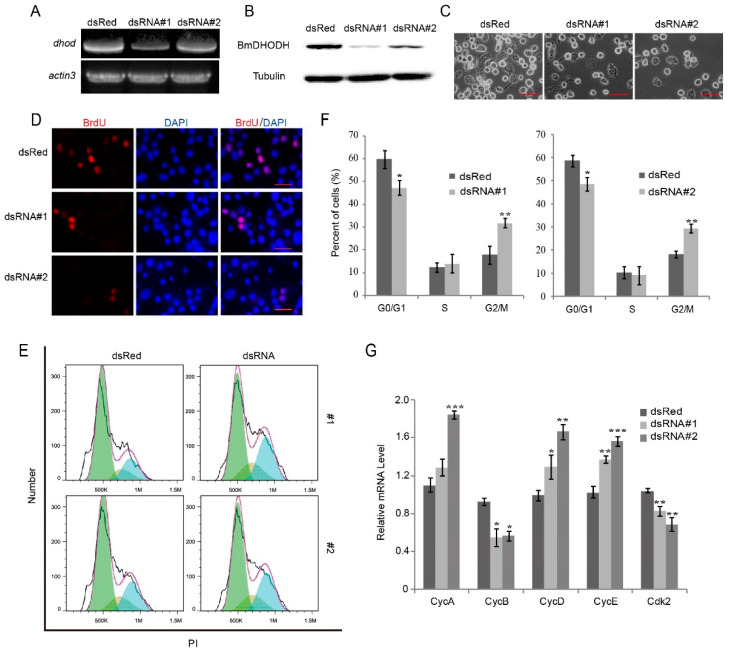
Knockdown of BmDHODH inhibited cell growth and proliferation. (**A**) RT-PCR was performed to detect *dhod* mRNA expression level in BmE-SWU3 cells after knockdown of BmDHODH by dsRNA interference for 48 h; *actin3* was used as a control. (**B**) Western blot assay was performed to detect BmDHODH protein expression level in BmE-SWU3 cells after knockdown of BmDHODH by dsRNA interference for 48 h; tubulin was used as a control. (**C**) Morphologic examination of BmE-SWU3 cells was shown after dsRNA interference for 48 h; dsRed was used as a control. Scale bar, 50 µm. (**D**) Cells were grown on coverslips after dsRNA interference for 48 h, respectively, and dsRed was used as a control. Cells were stained with an antibody against BrdU (red) and counterstained with 4′,6-diamidino-2-phenylindole (DAPI) (blue); scale bar, 100 µm. BrdU-positive cells were calculated randomly in at least 10 fields under microscopy. (**E**,**F**) Cell cycle was analyzed by a FACS assay, after knockdown of BmDHODH by dsRNA interference in BmE-SWU3 cells, and dsRed was used as a control. (**G**) qRT-PCR analysis was performed for BmE-SWU3 cells after dsRNA#1 or dsRNA#2 interference for 48 h, and dsRed was used as a control. In (**F**,**G**), data represent the average ± SD of at least three independent experiments. Statistical analysis was performed using Student’s *t*-test or one-way analysis of variance (ANOVA): * *p* < 0.05, ** *p* < 0.01, and *** *p* < 0.001.
